# Association of Serum PSP/REG Iα with Renal Function in Pregnant Women

**DOI:** 10.1155/2019/6970890

**Published:** 2019-04-21

**Authors:** Xiangyun Zhu, Beibei Dong, Theresia Reding, Youfan Peng, Hao Lin, Mengmeng Zhi, Manman Han, Rolf Graf, Ling Li

**Affiliations:** ^1^Department of Endocrinology, Zhongda Hospital, School of Medicine, Southeast University, Nanjing 210009, China; ^2^Department of Visceral and Transplantation Surgery, University Hospital of Zurich, Rämistrasse 10, Zurich 8091, Switzerland; ^3^Department of Clinical Science and Research, Zhongda Hospital, Southeast University, Nanjing 210009, China

## Abstract

Pancreatic stone protein/regenerating protein I*α* (PSP/REG I*α*) is a secretory protein produced in the pancreas, but its expression has also been observed in the kidney. It may be associated with kidney dysfunction. This study investigates the possible association between PSP/REG I*α* and kidney function in pregnant women. Serum PSP/REG I*α* levels were measured by a specific ELISA enzyme-linked immunosorbent assay. Maternal information and clinical and biochemical parameters were collected. Estimated glomerular filtration rate (eGFR) was calculated for all individuals to evaluate their renal function. Spearman's correlation and multiple linear regression analyses were performed to assess the associations between PSP/REG I*α* and eGFR, serum creatinine (Cr), blood urea nitrogen (BUN), and uric acid (UA). A total of 595 pregnant women were enrolled in the study. Participants with mildly reduced eGFR had higher PSP/REG I*α* levels [50.49 (35.02, 58.64)] than in the general population [26.84 (21.02, 33.07)] (p < 0.001). Included participants were stratified into PSP/REG I*α* quartiles; significant differences were observed in the levels of eGFR, serum Cr, BUN, and UA. PSP/REG I*α* was negatively correlated with eGFR (r = −0.402, p < 0.001) and positively associated with serum Cr (r = 0.468, p < 0.001), BUN (r = 0.166, p < 0.001), and UA (r = 0.207, p < 0.001). The linear regression analysis indicated that PSP/REG I*α* was associated with UA, BUN, and eGFR. High PSP/REG I*α* concentrations were closely associated with renal dysfunction in pregnant women. Our study provides clinical evidence that serum PSP/REG I*α* levels could be a novel biomarker for assessment of renal function in pregnant women.

## 1. Introduction

Chronic kidney disease (CKD) is a global public health concern because of its increasing incidence and prevalence. The prevalence of CKD is approximately 3% in women of childbearing age. Few studies investigated kidney disease during pregnancy [1, 2]. Women with renal disease are at a greater risk for maternal and fetal complications, including miscarriage, preterm delivery, preeclampsia, and fetal hypotrophy [3–5]. Moreover, pregnant women with a mild increase in glomerular filtration rate (GFR) possess a higher risk of adverse events than those without kidney disease [1, 6]. Although serum creatinine is a most important predictor for outcomes of pregnancy in CKD women, it is insensitive to mild and moderate renal impairment [7, 8]. Additionally, estimated GFR (eGFR) may likewise be unreliable in certain populations [9]. Given the scarcity of adequately sensitive and specific markers of kidney function in pregnant women, sensitive indicators should be identified to optimize clinical practice.

Regenerating (REG) family proteins are structurally similar proteins belonging to the calcium-dependent (C-type) lectin superfamily. In humans, REG family has five functional proteins, REG I*α*, REG I*β*, HIP/PAP, REG III, and REG IV. Pancreatic stone protein (PSP) is a product of REG I*α* gene; sequence comparison later revealed that REG I*α* and PSP proteins are identical [10]. Pancreatic stone protein/regenerating I*α* (PSP/REG I*α*) was originally identified as a 16 kDa polypeptide in pancreatic stones [10]. It has been studied mainly in the pancreas and was prominently upregulated in acute or chronic pancreatitis [11]. In addition, it appeared to promote regeneration of islets by induction of cellular proliferation [12]. PSP/REG I*α* was also found in the urine and renal calculi of healthy individuals [13, 14], suggesting a physiological role of PSP/REG I*α* in the kidney. Sobajima et al. [15] reported that urinary PSP/REG I*α* was markedly elevated in patients with various renal diseases, including diabetic nephropathy. Our previous studies have accumulated compelling evidence for elevated PSP/REG I*α* levels in patients with chronic diabetic complications and diabetic kidney disease [16, 17]. Nevertheless, limited information is available regarding the association between serum PSP/REG I*α* and renal function in pregnant women. Identification of such a serologic marker may provide new approaches for the prevention of progressive renal insufficiency in pregnancy. Therefore, we aimed to investigate the association between serum PSP/REG I*α* levels and renal function in pregnant women.

## 2. Materials and Methods

### 2.1. Study Subjects

This cross-sectional study of 595 pregnant women was conducted at the Zhongda Hospital Affiliated with Southeast University in Nanjing, China. The study was approved by the hospital ethics committee (2016ZDSYLL076-P01), and experimental methods were performed strictly in accordance with the approved guidelines. Written informed consent was obtained from all participants.

Pregnant women were recruited before their delivery from March 2016 to December 2017. Pregnant women aged 20–55 years were included in the study. The exclusion criteria included the following: maternal age of less than 20 years and more than 55 years, diagnosis of pregestational diabetes, autoimmune diseases prior to pregnancy, prepregnancy cardiovascular diseases, and severe hepatic disorders before pregnancy.

### 2.2. Data Collection and Quality Assessment

We collected information on demographics, maternal age, education and employment, pregestational weight and height, last menstruation, pregnancy and obstetrical history, hypertension, and macrosomia in previous pregnancies. From each pregnant woman, 5 ml of peripheral blood was collected and centrifuged directly for 6 min at 3,000 g. The obtained serum was immediately frozen in sterile tubes at −80°C. Other clinical biochemical parameters, such as fasting blood glucose (FBG), total cholesterol, triglyceride, low-density lipoprotein, high-density lipoprotein, Cr, uric acid (UA), and blood urea nitrogen (BUN), were measured using the standard methods. The Laboratory Center of Zhongda Hospital conducts internal and external quality control procedures directed by the Chinese Laboratory Quality Control. Body mass index (BMI) was calculated using the following formula: BMI = body weight (kg)/body height (m^2^). eGFR was calculated using the modified CKD Epidemiology Collaboration (CKD-EPI) equation for Asians [18]. The following formula was used: GFR (ml/min/1.73 m^2^) = 141 × min (SCr/0.7, 1)^−0.329^  × max (SCr /0.7, 1)^−1.209^  × 0.993^age^  × 1.018. Kidney function was classified, using the method proposed by the National Kidney Foundation, into three categories: normal (eGFR ≥ 90 ml/min/1.73 m^2^), mildly reduced (eGFR = 60 ml/min/1.73 m^2^ to 89 ml/min/1.73 m^2^), and moderately–severely reduced (eGFR = 15 ml/min/1.73 m^2^ to 59 ml/min/1.73 m^2^) [8].

### 2.3. PSP/REG I*α* Enzyme–Linked Immunosorbent Assay (ELISA)

PSP/REG I*α* level was determined using an isoform specific ELISA, as previously described [19]. The serum collected from patients was incubated in plates precoated with guinea pig anti-human recombinant PSP/REG I*α* antibody. Rabbit anti-human PSP/REG I*α* was added to the plate which was then subjected to detection with phosphatase–conjugated anti-rabbit immunoglobulin G. The detection limit was < 0.1 ng/ml, and the interplate variance was < 10%. The antibodies used in this assay are specific for human PSP/REG I*α* with no known cross reactivities to recombinant human REG proteins. The PSP/REG I*α* ELISA is covered by a patent governed by international law.

### 2.4. Statistical Analysis

Statistical analyses were performed by SPSS 19.0 software. Continuous variables were indicated as means ± SDs if the continuous variables followed normal distribution. Nonnormally distributed values were presented as median (interquartile range [IQR]). Significant differences were assessed by either the Wilcoxon–Mann–Whitney test or one-way ANOVA. Correlations between PSP/REG I*α* and clinical parameters were determined using Pearson's correlation analysis or Spearman's rank correlation coefficient, whichever was appropriate. Multiple linear regression analysis was also performed to assess the potential association between PSP/REG I*α*, as dependent variable, and the value of each kidney function indicator by entering age, BMI, and FBG, as independent variables in the model. All tests were two-sided, and values at p < 0.05 were considered statistically significant.

## 3. Results

### 3.1. Description of the Study Population

All of the initial 597 participants were subjected to PSP/REG I*α* measurement, and about 595 of them were analyzed for serum Cr, BUN, and UA. Finally, 595 pregnant women were included in the study. Analysis with the CKD-EPI equation indicated that 583 participants (97.98%) had normal eGFR and 12 participants (2.02%) had mildly reduced eGFR. In the total population, the median (IQR) concentration of PSP/REG I*α* was 26.95 (21.24–33.28) ng/ml, and the participants with mildly reduced eGFR had higher PSP/REG I*α* levels [50.49 (35.02, 58.64) ng/ml] than in the general population [26.84 (21.02, 33.07) ng/ml]. Subsequently, the study population was stratified into PSP/REG I*α* quartiles (Table 1). Pregnant women in quartile IV showed significantly a higher probability of being nullipara of the four groups (p < 0.05). The distribution values of serum Cr, eGFR, BUN, and UA showed significant differences among the quartiles. Moreover, the eGFR significantly decreased from the bottom to the top quartile of PSP/REG I*α*. The median values of serum Cr, BUN, and UA significantly increased among the PSP/REG I*α* categories. No significant differences were observed in the value distribution of the other parameters.

### 3.2. Relationships of PSP/REG I*α* Levels to Kidney Function Indicators in Pregnant Women

**Figure 1:**
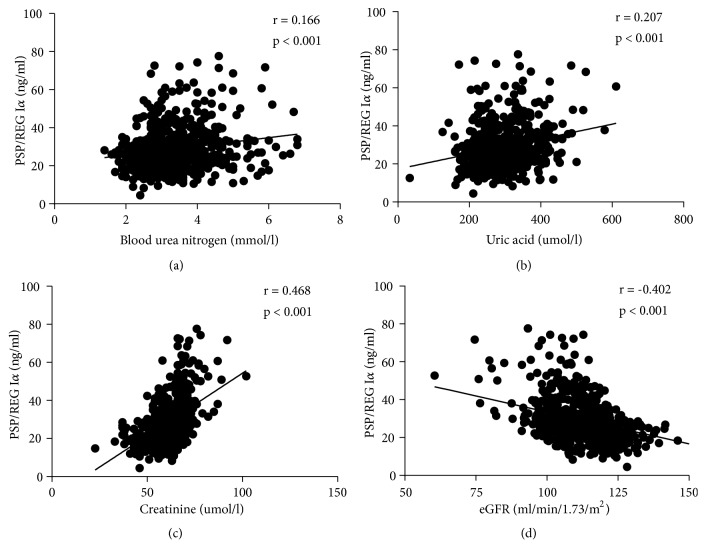
Relationships of serum PSP/REG I*α* levels with kidney function in pregnancy women. (a) Correlation of PSP/REG I*α* with blood urea nitrogen (r = 0.166, p < 0.001). (b) Correlation of PSP/REG I*α* with serum uric acid (r = 0.207, p < 0.001). (c) Correlation of PSP/REG I*α* with creatinine (r = 0.468, p < 0.001). (d) Correlation of PSP/REG I*α* with eGFR (r = -0.402, p < 0.001).

Considering the significant differences in the value distribution of kidney function indicators, we analyzed their correlation with PSP/REG I*α*. Spearman's correlation analysis showed that serum PSP/REG I*α* levels were negatively correlated with eGFR (r = −0.402; p < 0.001) and positively associated with serum Cr (r = 0.468, p = 0.001), BUN (r = 0.166, p < 0.001), and UA (r = 0.207, p < 0.001) (Figure 1). In addition, a partial correlation was found between PSP/REG I*α* level and eGFR after adjusting for UA and BUN levels. A moderate correlation was also observed between PSP/REG I*α* and eGFR (r = −0.330, p < 0.001).

To further investigate the correlation between PSP/REG I*α* and renal function indicators, we performed subgroup analysis according to PSP/REG I*α* quartiles. PSP/REG I*α* was moderately correlated with Cr in quartiles III (r = 0.237, p = 0.004) and IV (r = 0.412, p = 0.009) and with eGFR in quartiles III (r = −0.213, p < 0.001) and IV (r = −0.355, p < 0.001) (Table 2).

### 3.3. Multiple Linear Regression Model

Age, BMI, FBG, UA, BUN, and eGFR were used in the multiple regression equation. In this model, UA (*β* = 0.022, p = 0.001), BUN (*β* = 1.040, p = 0.036), and eGFR (*β* = -0.288, p < 0.001) were all independent predictors of PSP/REG I*α*. Participants with elevated UA and BUN and decreased eGFR would have higher PSP/REG I*α* concentrations (Table 3).

## 4. Discussion

The present study analyzed associations between PSP/REG I*α* and renal function in a cross-sectional study of 595 pregnant women. To date, such associations have not been clearly understood. We revealed that high PSP/REG I*α* concentrations exhibit moderate association with unfavourable renal function in pregnant women. This study provides clinical evidence that serum PSP/REG I*α* level may be a suitable marker for assessment of renal function in pregnant women.

Studies of kidney disease during pregnancy classify women primarily on the basis of their serum Cr levels. However, the accuracy of using serum Cr level as a marker of renal function is low due to its nonlinear association with eGFR; that is, such association varies with age, sex, race, and lean body mass [7]. A previous study categorized CKD severity according to eGFR [20]. Although eGFR is the most important predictor of pregnancy outcomes in women with kidney disease, it is insensitive to mild and moderate renal impairment. It is unlikely that any single biomarker is perfect in the diagnosis of pregnant women's diseases, but detection of multiple biomarkers and statistical analysis markedly enhance the diagnostic sensitivity and specificity. In the present study, we found that PSP/REG I*α* may be a suitable auxiliary marker of a decline in renal function in pregnant women.

Initially, PSP and REG I*α* were discovered independently in the fields of pancreatitis [21] and diabetes [22]. Sequence analysis later revealed that PSP and REG I*α* are indeed identical, thus suggesting that the combined terms of PSP/REG I*α* should be used in the future [23]. PSP/REG I*α* mRNA is mainly detected in the pancreas, but its expression has also been found in the gastric mucosa and the kidneys [21, 24]. The REG receptor (EXTL3) mRNA expression has also been detected in the liver, heart, kidney, spleen, thymus, stomach, small intestine, colon, pancreatic acinar and ductal cells, adrenal gland, pituitary gland, testis, and brain [25–27], suggesting the possible involvement of the REG-REG receptor signal system in a variety of cell types other than *β*-cells. In our previous study, PSP/REG I*α* levels showed consistent positive correlation with serum Cr and UA levels and negative correlation with eGFR; hence, serum PSP/REG I*α* levels closely reflect glomerular injury and declining renal function in patients with type 2 diabetes [16]. All these results support that PSP/REG I*α* may be associated with kidney disease. The present study confirmed that PSP/REG I*α* is a relatively a reliable and useful marker for assessment of renal function in pregnant women.

Three mechanisms have been suggested to explain the relationship of PSP/REG I*α* and kidney function. Firstly, since PSP/REG I*α* is a low molecular weight protein of 16 kd, it crosses the glomerular basement membrane and undergoes reabsorption in the proximal renal tubules [15]. A correlation was also observed between eGFR and PSP/REG I*α* levels; therefore, the increase in PSP/REG I*α* is more likely to reflect reduced glomerular filtration capacity rather than reabsorption from damaged renal tubules. Another possible mechanism is that PSP/REG I*α* may be involved in the kidney development. PSP/REG I*α* was first identified in the pancreas as an anti-stone forming molecule [28, 29]. However, it also has a function as a growth factor for the pancreas. As PSP/REG I*α* is predominantly expressed in the pancreas, it may be trapped in the kidney, and the kidney may not produce it [24]. There are reports that PSP/REG I*α* has been identified in the human kidney and urine also, which is elevated in diabetic patients [15], suggesting that it may be involved in diabetic kidney hypertrophy as a kidney growth factor. And finally, it has been repeatedly shown that advanced CKD is associated with a state of chronic inflammation, as evidenced by either elevated levels of various proinflammatory cytokines (IL-1*β*, IL-6, TNF-*α*, etc.) or altered levels of acute-phase proteins (CRP, albumin, fetuin-A, etc.), which in turn are associated with increased rate of CKD progression and risk of death [30, 31]. PSP/REG I*α* serves as an inflammatory factor that may be involved in CKD.

We found reliable interactions between increased PSP/REG I*α* values and low eGFR. Based on Spearman's rank correlation analysis, PSP/REG I*α* levels showed consistent positive correlations with serum Cr, BUN, and UA levels and negative correlation with eGFR. Thus, serum PSP/REG I*α* levels closely reflect glomerular injury and decline in renal function in pregnant women. UA is predominantly cleared by the kidneys; as such, decline in eGFR will be almost universally associated with increased UA [32]. Hence, UA was considered primarily a marker of kidney damage and a secondary independent risk factor for kidney disease development and progression. Our findings on the correlation between PSP/REG I*α* and UA levels further imply that the protein is associated with kidney damage.

When we used eGFR to define mildly reduced eGFR and normal groups, we found that participants with mildly reduced eGFR have high PSP/REG I*α* levels. Moreover, PSP/REG I*α* was moderately correlated with serum Cr and eGFR only in the subgroups of quartiles III and IV. It is reasonable that both PSP/REG I*α* and eGFR are at normal levels and irrelevant when kidney function is normal. In addition, elevated PSP/REG I*α* concentrations are sensitive to renal dysfunction. Overall, PSP/REG I*α* is associated with clinical kidney function impairments detected using eGFR. To further investigate the correlation between PSP/REG I*α* and renal function, we conducted multivariate linear regression. eGFR, BUN, and UA remained significantly associated with PSP/REG I*α* even after adjusting for age, BMI, and FBG. This result confirms that PSP/REG I*α* may be a useful epidemiological tool for evaluating mild kidney impairment in pregnant women. However, after adjusting for serum Cr, the association between PSP/REG I*α* and eGFR was no longer statistically significant because the CKD-EPI equation used to estimate log GFR includes log serum Cr.

To our knowledge, this work is the first to investigate the correlation between serum PSP/REG I*α* and renal function in pregnancy. This work also conducted comprehensive measurement of potentially confounding variables. However, this study has several limitations. First, the findings are limited to cross-sectional assessment. Further research must confirm if the relationship between PSP/REG I*α* and eGFR is causal or an epiphenomenon. Second, our sample size of pregnant women with stage 3 or 4 CKD was relatively small due to the nature of the research question. Further prospective studies should recruit patients with different stages of CKD to determine the potential of PSP/REG I*α* as a biomarker for diagnosis and evaluation of the onset and development of CKD in pregnant women.

## 5. Conclusions

In conclusion, a high PSP/REG I*α* level is associated with kidney function markers, namely, eGFR and serum levels of Cr, BUN, and UA. Our study provides clinical evidence that serum PSP/REG I*α* levels may be a suitable marker for assessment of renal function in pregnancy.

## Figures and Tables

**Table 1:** Maternal characteristics and clinical characteristics stratified according to quartiles of PSP/REG I*α* in pregnant women.

Quartiles of PSP/REG I*α*	I (n=148)	II (n=149)	III (n=149)	IV (n=149)	*p*-value
Maternal age (years)	30.96 ± 4.54	30.59 ± 4.80	30.77 ± 4.70	31.122 ± 4.85	0.781
Education (years)	14.88 ± 2.29	14.88 ± 2.70	15.336 ± 2.07	15.07 ± 2.47	0.304
≤ 12^a^	14(9.40)	15(10.00)	39(26.17)	41(27.52)	
12–16^b^	122(81.88)	129(86.00)	100(67.11)	88(59.06)	
> 16	13(8.72)	6(4.00)	11(7.38)	19(12.75)	
Nullipara [n (%)]	67(44.97)	74(49.66)	61(40.94)	84(56.38)	0.001
Pregestational BMI (kg/m^2^)	21.62 ± 3.60	21.91 ± 3.01	21.21 ± 2.80	21.71 ± 3.22	0.277
Family history of hypertension [n (%)]	11(7.38)	11(7.33)	20(13.42)	12(8.05)	0.196
Family history of diabetes [n (%)]	8(5.37)	4(2.67)	4(2.67)	9(6.04)	0.321
Systolic BP (mmHg)	116.42 ± 10.31	116.49 ± 11.26	116.80 ± 9.58	118.32 ± 13.12	0.415
Diastolic BP (mmHg)	73.40 ± 8.80	73.07 ± 8.09	72.73 ± 7.09	74.07 ± 10.05	0.572
FBG (mmol/L)	4.54 ± 0.81	4.56 ± 0.98	4.51 ± 0.84	4.57 ± 0.98	0.936
Total Cholesterol (mmol/L)	6.42 ± 1.15	6.65 ± 1.11	6.82 ± 1.37	6.72 ± 1.37	0.163
Triglycerides (mmol/L)	4.22 ± 1.58	4.11 ± 1.53	4.32 ± 1.83	4.68 ± 2.10	0.132
LDL-cholesterol (mmol/L)	3.29 ± 0.84	3.51 ± 0.88	3.59 ± 1.10	3.51 ± 1.00	0.181
HDL-cholesterol (mmol/L)	2.06 ± 0.58	2.00 ± 0.34	2.00 ± 0.35	1.97 ± 0.38	0.585
Baseline renal parameter					
UA (*μ*mol/L)	273.86 ± 64.48	287.34 ± 60.99	292.34 ± 58.81	318.52 ± 84.61	0.001
Creatinine (*μ*mol/L)	56.81 ± 8.52	58.95 ± 7.79	61.25 ± 7.35	64.91 ± 8.36	0.001
BUN (mmol/L)	3.25 ± 0.85	3.39 ± 0.95	3.34 ± 0.89	3.65 ± 0.87	0.001
eGFR (ml/min/1.73* *m^2^)	118.78 ± 17.85	116.04 ± 9.66	113.29 ± 9.85	107.99 ± 11.43	0.001

Significance, *p *<* *0.05. Data are presented as *n* (%), mean ± SD, or median (interquartile range) as appropriate. ^a^Accepted education for 12 years means already obtained the degree of high school. ^b^Accepted education for 16 years means already obtained the degree of bachelor.

**Table 2:** Association between serum PSP/REG I*α* and kidney function indicator by quartiles of PSP/REG I*α*.

	I (n=148)		II (n=149)		III (n=149)		IV (n=149)	
	*r*	*p*	*r*	*p*	*r*	*p*	*r*	*p*
UA	0.191	0.020	0.021	0.804	-0.095	0.247	0.100	0.224
Creatinine	0.177	0.051	-0.044	0.593	0.237	0.004	0.412	0.001
BUN	0.143	0.083	-0.090	0.276	0.162	0.048	0.059	0.478
eGFR (ml/min/1.73* *m^2^)	-0.124	0.132	0.079	0.340	-0.213	0.009	-0.355	0.001

Significance, p* *<* *0.05.

**Table 3:** Multiple linear regression analysis between PSP/REG I*α* and eGFR in pregnant women.

Variables analyzed	*β*	SE of *β*	Standardized *β*	T	*p*
Age	0.112	0.091	0.046	1.223	0.222
BMI	-0.078	0.128	-0.023	-0.610	0.542
FBG	0.033	0.477	0.003	0.070	0.945
BUN	1.040	0.496	0.082	2.099	0.036
UA	0.022	0.007	0.134	3.338	0.001
eGFR	-0.288	0.034	-0.334	-8.510	0.001

Significance, *p *<* *0.05. Abbreviations: *β*, regression coefficient; SE, standard error; T, One-Sample T Test; BMI, body mass index; FPG, fasting plasma glucose; BUN, blood urea nitrogen; UA, uric acid; eGFR, estimated glomerular filtrations rate.

## Data Availability

The data used to support the findings of this study are available from the corresponding author upon request.

## Abbreviations

CKD: Chronic kidney disease
PSP/REG I*α*: Pancreatic stone protein/regenerating protein I*α*
eGFR: Estimated glomerular filtration rate
Cr: Serum creatinine
BUN: Blood urea nitrogen
UA: Uric acid
CRP: C-reactive protein
IL-6: Interleukin 6
TNF-*α*: Tumor necrosis factor *α*
FBG: Fasting blood glucose
BMI: Body mass index
Systolic BP: Systolic blood pressure
Diastolic BP: Diastolic blood pressure
TC: Total cholesterol
TG: Triglyceride
HDL: High-density lipoprotein
LDL: Low-density lipoprotein
r: Correlation coefficient.
